# Characterization and Performance Evaluation of Liquid Biodegradable Mulch Films and Its Effects on Peanut Cultivation

**DOI:** 10.3390/polym16172487

**Published:** 2024-08-31

**Authors:** Jie Shi, Shaoli Wang, Zhongxue Yang, Baoyan Li, Ruijue Chen, Fanzhi Bu, Binghui Luan, Baoyou Liu, Peiqiang Li

**Affiliations:** 1 Yantai Academy of Agricultural Sciences, Yantai 265500, China; zhibaoshijie@163.com (J.S.); shaoliwang123@126.com (S.W.); byli1314@163.com (B.L.); lbh815@163.com (B.L.); 2 College of Chemistry and Material Science, Shandong Agricultural University, 61 Daizong Road, Taian 271018, China; zxyang@sdau.edu.cn (Z.Y.); rjchen6310@163.com (R.C.); 17662540427@163.com (F.B.)

**Keywords:** liquid biodegradable mulch films, characterization, performance, soil conditions, peanut growth, peanut yield

## Abstract

With the development of material science and increasing awareness of ecological environmental protection, liquid biodegradable mulch films (LBDMs) have garnered significant public interest. In this research, new LBDMs were developed using hydrophobically modified polymer materials, surfactants, and photosensitive catalysts. Characterization by scanning electron microscopy (SEM) revealed good material compatibility. LBDMs exhibited excellent wettability and degradability, effectively covering soil surfaces and enhancing soil moisture conservation, with a degradation rate of 76.09% after 80 days of burial. The field performance experiment was conducted over two consecutive years, 2021 and 2022, to assess differences in soil temperature and moisture, peanut agronomic traits, pod traits, and yield under four treatments: non-mulching (CK), LBDMs, clear polyethylene mulch films (CPEMs), and black polyethylene mulch films (BPEMs). LBDMs increased soil temperature by 0.56 °C and soil moisture by 19.25%, accelerated the seedling stage by 4-to-6 days, and improved the average emergence rate by 15.91%. Furthermore, LBDMs significantly promoted peanut growth, and it increased yield by 14.34% compared to CK. LBDMs performed comparably to the two types of PE films in maintaining soil conditions and different crop phenotype traits, including plant height, branch number, yield, and quality, and they even outperformed PE films in productivity per plant and 100-kernel weight. These findings suggest that LBDMs are a promising eco-friendly alternative to traditional PE films.

## 1. Introduction

The peanut (*Arachis hypogaea* L.) is a significant grain and oil crop in China, with its yield ranking first globally [1]. For over half a century, polyethylene (PE) mulch films have been widely used in agriculture to enhance peanut productivity [2]. Film mulching can elevate soil temperature, maintain soil moisture, suppress weed growth, reduce the occurrence and spread of phytopathogens, and enhance crop growth and yield [2,3,4,5]. However, the extensive use of PE mulch films has led to severe agricultural non-point source pollution with long-lasting ecological impacts [6,7]. Residual plastic fragments in the soil degrade the structure of the plow layer, impede water and fertilizer transport, hinder soil microorganism activity, and eventually cause soil compaction, which negatively affects crop growth [8,9]. To promote sustainable agricultural practices, biodegradable films have emerged as a research focus. These films offer similar warming and moisture conservation benefits as conventional PE films and often surpass PE films in improving soil properties and crop growth [10,11]. Biodegradable films naturally degrade through microbial action and finally break down into CO_2_ and H_2_O [12,13]. Therefore, biodegradable films represent a promising alternative to traditional PE films in agricultural ecosystems [14,15].

In recent years, researchers have proposed the feasibility of using LBDMs as substitutes for traditional plastic films. Various research institutions, both in China and abroad, have studied and applied LBDMs [16,17,18,19]. These films are emulsion suspensions with organic polymers as the main carbon skeleton, significantly reducing labor intensity and improving efficiency compared to the manual application of plastic films [17]. LBDMs exhibit excellent wettability, forming a multi-molecular network of gelatinous film after spraying on the soil surface [20]. Spraying LBDMs binds soil particles together, forming an aggregate structure that effectively preserves soil temperature and moisture, while reducing water evaporation without impeding water infiltration [21]. And then, it promotes the growth and yield of crops [22,23]. Additionally, integrating water-soluble fertilizers and pesticides beneficial to crops into the LBDM system creates a multifunctional film, further enhancing its value by reducing labor intensity through combined applications [4,7]. However, existing LBDMs are typically composed of chemical polymers with poor degradability or highly hydrophilic materials such as humic acid, starch, cellulose, ethyl cellulose, and polyglutamic acid, or simply mixed with these materials [5]. These compositions are either not environmentally friendly or fail to form a durable film, resulting in a poor performance characterized by inelasticity, fragility, and susceptibility to rainfall erosion, with a relatively short effective duration [24,25,26,27]. Currently, the research and development of LBDMs are still in the small-scale experimental stage, with significant challenges remaining before large-scale adoption can be achieved. Therefore, developing a high-performance, production-suitable liquid biodegradable mulch film has become an urgent need in this field.

In this study, newly developed LBDMs were prepared using hydrophobically modified polymer materials, surfactants, and photosensitive catalysts. These films exist in liquid form and can be directly sprayed onto the soil to form a biodegradable film. The use of hydrophobically modified polymer materials enhances the film’s erosion resistance and improves soil water retention. To determine whether LBDMs can meet the varying soil condition requirements at different crop growth stages, we characterized their performance and selected peanuts as a model crop to evaluate their effects on crop growth and yield. The objective of this study is to verify the superior performance of LBDMs, including their wettability, degradability, temperature regulation, water retention capabilities, etc. Additionally, we aim to explore the impact of LBDMs on the physiological traits, quality, and yield of peanuts in the Yantai area, guiding the application of LBDMs in Yantai and surrounding regions.

## 2. Materials and Methods

### 2.1. Experimental Site

The field experiments were conducted at the experimental plot of Yantai Academy of Agricultural Sciences (37°29′ N, 121°16′ E), Yantai, Shandong Province, China, in 2021 and 2022. The soil was loam, and the plot was flat with medium and uniform fertility. The contents of hydrolyzable nitrogen, available potassium, and available phosphorus at 0~20 cm depth were 32.61~39.03 mg·kg^−1^, 183.69~188.53 mg·kg^−1^, and 111.34~126.96 mg·kg^−1^, respectively. The content of organic matter was 1.68~1.83%, and the soil pH value was 6.40~6.66. In the study area, the climate was the temperate monsoon with four distinctive seasons, sufficient sunshine, and moderate rainfall with a mean annual temperature ranging from 12.7 °C to 13.0 °C. The annual rainfall was from 830.6 mm to 989.9 mm, of which 70~90% fell in a major part of the growing season between June and September. The rainfall and the air temperature during the experimental period were measured using an automatic weather station (RS-ECTH-N01-TR temperature and humidity sensors, Jinan, China; ZQZ-A automatic weather station, Beijing, China) at the experimental site.

### 2.2. Field Experimental Design and Treatments

The big-fruit-type peanut variety ‘Huayu 22’, provided by Shandong Peanut Research Institute and approved by the Shandong Province Crop Variety Approval Committee in February 2003, was selected in this study. This variety is an early-maturing ordinary peanut, with high quality, high yield potential, high stress resistance, moderate disease resistance, and a 130-day growth period [28].

Four treatments were designed and applied: (1) peanut cultivation mulched with liquid biodegradable mulch films (LBDMs), (2) peanut cultivation mulched with black polyethylene mulch films (BPEMs), (3) peanut cultivation mulched with clear polyethylene mulch films (CPEMs), and (4) peanut cultivation with no mulching as the control (CK). Each treatment was replicated three times, a total of 12 plots, and each plot area measured 42 m^2^ (21 m × 2 m) in a randomized block arrangement. Each plot contained 2 ridges, and 2 rows were planted in each ridge. The plant spacing was 20 cm, the ridge length was 21 m, and 2 seeds were sown in each hole. A 0.8 m wide border was set between each plot for field management and sampling activities. All the polyethylene (PE) film mulches were 0.01 mm thick and 130 cm wide (Yantai Changsheng Plastic Factory, Yantai, China). With PE film mulching, the films were used flat to cover the surface of the ridges, where the film edges were covered carefully and compacted with soil. LBDMs were sprayed evenly on the soil surface, with the help of the knapsack sprayer, and the spraying range was 1 m. The seed cultivar ‘Huayu 22’ was sown at a rate of 420 per plot using a handheld hole-sowing machine, with a sowing depth of 4~5 cm. The seeding and film mulching were conducted on 3 May and 8 May, and the peanuts were harvested on 13 September and 16 September, in 2021 and 2022, respectively.

Herbicides were applied before sowing, and weeds were manually controlled during the crop growth period. Before the experiment, deep plowing to a depth of approximately 20 cm was performed using a tractor-mounted moldboard plow. No additional tillage or irrigation was conducted during the entire experimental period.

### 2.3. Preparation and Characterization of LBDMs Mulching Films

#### 2.3.1. Preparation of LBDMs

A certain amount of polymer materials chitin (0.72 wt%), polycarbonate (1.52 wt%), alkyl glucoside (0.06 wt%), coconut oil-based glucoside (0.06 wt%), cellulose acetate (0.85 wt%), and carboxymethyl cellulose (0.85 wt%) were weighed and mixed with water at a mass ratio (solid/liquid) of 1:2 [18,29,30]. They were heated in water to 50~60 °C and dispersed. Carboxylic acids, epoxy compounds, halogenated hydrocarbons, aliphatic acyl chloride, and isocyanate were added as hydrophobic reagents (0.68 wt%) for hydrophobic modification, and amine compounds (0.34 wt%) were used as an end-capping reagent to obtain hydrophobically modified polymer materials [31,32,33]. Then, the above-mentioned hydrophobically modified polymer material was weighed, and the surfactant and photosensitive catalyst were added and stirred with water to obtain a gelatinous viscous transparent liquid film. The composition ratio of surfactant, photo-sensitive catalyst, and hydrophobically modified polymer material was 0.05:0.02:1. The mass ratio of the above surfactants, including calcium dodecyl benzene sulfonate and styrene phenol polyoxyvinyl ether, is 1.5:1. The photosensitive catalyst was metal porphyrin [34]. The prepared mixture was poured into culture plates and dried under natural conditions, and then the films were uncovered and reserved as spares.

#### 2.3.2. Characterization of LBDMs

The surface morphology and microstructure of the LBDMs sample films were observed using a scanning electron microscope (SEM) (Phenom Pure, The Netherlands) at various magnifications (Mag = 500×, 1500×, 3000×, and 13,000×). Before testing, the LBDM samples were dried in a 60 °C drying oven for 12 h to form thin-film samples. These samples were then affixed to the test bench with a conductive tape. The samples were sputter-coated with gold and subsequently imaged using the SEM with an accelerating voltage of 10 kV.

#### 2.3.3. LBDMs Wettability

To evaluate the wettability of LBDMs, the contact angle between LBDMs and soil surface was measured by a simplified sessile drop method at room environment (25 ± 2 °C, 50% RH) [35,36]. Four treatments were set up according to the soil granularity size in this experiment, and an LBDMs droplet with a volume of 15 μL was deposited onto the soil surface. The contact angle and the complete infiltration time of each treatment were recorded, and subsequently the wettability of LBDMs was characterized under a microscope. In addition, the soil collected from the field was placed in a petri dish, and the liquid film was sprayed evenly on the surface of the soil. The treatment without spraying liquid film was used as the control. After the film was formed by natural drying, a certain amount of water was sprayed on the surface of the soil, so that the soil was completely immersed in water to simulate the natural rainwater soaking process. After seven days, the effect of water immersion on the stability of the liquid film was observed.

#### 2.3.4. LBDMs Degradation

The degradable property of LBDMs was investigated by the soil burial test method [37]. After drying the LBDMs to form solid films, three kinds of films (LBDMs, CPEMs, and BPEMs) were cut into a square of 3 cm × 3 cm, buried in the soil at a depth of 10 cm, and maintained by 30–40% soil moisture. Within 80 days after burial, the films were taken out every 10 days, and the degradation situation was observed. The surface morphology of the sample films after soil burial degradation was analyzed using the SEM characterization method described in Section 2.3.2. In addition, to determine the degradation rate of LBDMs, the film samples, before being buried with soil, were weighed, and then they were taken out every 10 days, washed with distilled water, dried at 40 °C for 6 h, and weighed again. Each treatment was repeated three times to take the average value, and the mass loss was obtained. The degradation rate of LBDMs was calculated according to the following formula:

Degradation rate (%) = (mass before degradation − mass after degradation)/mass before degradation × 100

### 2.4. Determination of Soil Temperature and Moisture

After sowing, the soil temperature and moisture of the different treatments at 10 cm depth were measured with the 485-type soil temperature and moisture sensor (RS-ECTH-N01-TR; Shandong Renke Measurement and Control Technology Co., Ltd., Jinan, China), and the temperature and moisture were recorded every 2 h to see the detailed changes until the end of the whole growth period.

### 2.5. Peanut Growth and Developmental Progress and Yield

#### 2.5.1. Seedling Emergence

The seedling stage and emergence rates for the different treatments were recorded after sowing. During this period, the number of successfully emerged seedlings in all test plots was recorded daily. The seedling emergence rate was calculated by the formula as follows. When the seeding emergence rate reached 50%, that day was determined as the seeding stage.

Seedling emergence rate (%) = (the number of successful seedling emergence/the number of total seeds) × 100

#### 2.5.2. Growth Parameters of Peanuts

Each growth stage of the peanut was recorded. During the seedling stage, anthesis stage, pod bearing stage, and harvesting stage, the values of plant height were recorded every 10 days until they were seriously lodging and could not be measured. Fifteen peanut plants were continuously selected from the middle ridge of each test plot (a total of forty-five plants for every treatment) to record the total branch number and lateral branch length.

#### 2.5.3. Determination of Peanut Pod Traits

To determine peanut pod traits, fifteen peanut plants were continuously selected from the middle ridge of each replicate (repeat three times, a total of forty-five plants for every treatment), and all peanut pods were collected from selected plants. Total pod number, full pod number, immature pod number, total kernel number, germinated kernel number, single-kernel pod number, and double-kernel pod number were recorded. The full pod rate, immature pod rate, kernel rate, single kernel rate, double kernel rate, and germinated kernel rate were calculated for each treatment.

#### 2.5.4. Peanut Yield

During the harvest period, 2 sample points were randomly selected from each plot, in which both ends were removed, and a 1 m^2^ area with uniform growth of each sample point was harvested to determine the final pod yield. The pod yield for each experimental plot was determined by fresh weight and was used to calculate yield per hectare. ‘Huayu 22’ groundnut is usually harvested at about 130 days in the eastern part of Shandong, and the final harvest date was determined based on visual observations of leaf senescence and peanut kernel maturity. At the same time, fifteen peanut plants were continuously selected from the middle ridge of each replicate (repeat three times, a total of forty-five plants for every treatment) to count the productivity per plant, 100-kernel weight, and 100-pod weight. Among them, the determination of single plant productivity required the mature pods to be fully sundried and weighed to calculate the average weight of the pods per plant.

### 2.6. Statistical Analyses

The effects of the treatments on the measured parameters were evaluated using one-way ANOVA from the SAS package, and the least significant difference (LSD) was used to compare means [38]. In all cases, differences were deemed to be significant if *p* < 0.05. Data graphs were generated using GraphPad Prism 8.0.2 software.

## 3. Results and Discussion

### 3.1. Weather Conditions

**Figure 1:**
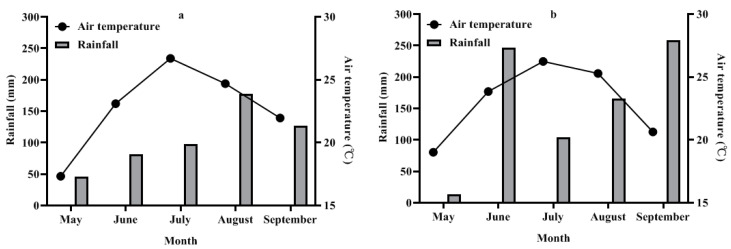
Monthly air temperature and rainfall distribution were monitored throughout the experimental periods in 2021 (**a**) and 2022 (**b**).

The weather conditions, including rainfall and air temperature, varied between the two growing seasons of peanuts (Figure 1). From May to September in 2021, air temperatures ranged from 9.9 °C to 28.8 °C, with a total rainfall of 530.2 mm and a mean monthly rainfall of 106.0 mm. The highest rainfall occurred in August. In contrast, during the same period in 2022, temperatures ranged from 11.8 °C to 31.5 °C, with a total rainfall of 788.2 mm and a mean monthly rainfall of 157.6 mm. The maximum rainfall was recorded in September. These differences in rainfall and air temperature between the two years were expected to influence peanut development and result in yield variations.

### 3.2. LBDMs Surface Morphologies, Wettability, and Degradability

#### 3.2.1. LBDMs Surface Morphologies

**Figure 2:**
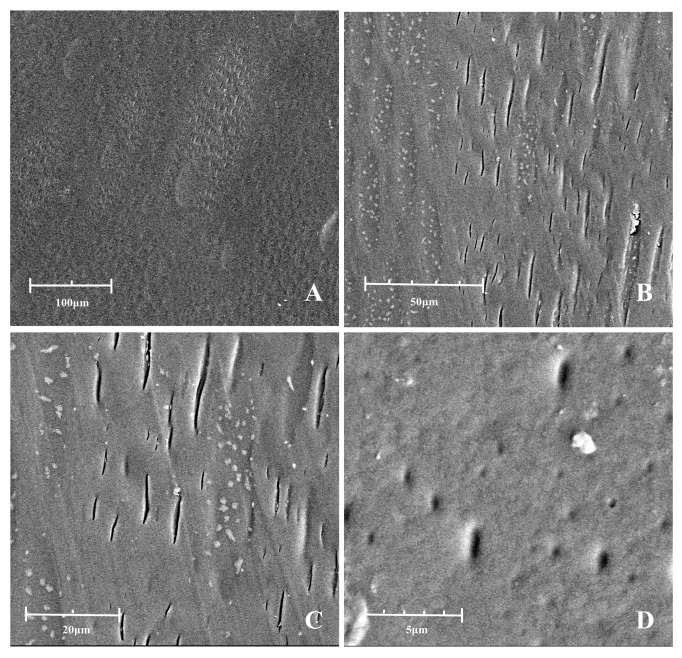
SEM images of the LBDMs surface morphology at different magnifications. (**A**) Mag = 500×. (**B**) Mag = 1500×. (**C**) Mag = 3000×. (**D**) Mag = 13,000×.

To visually assess the surface morphology and dispersion of LBDMs, the dried films were observed using scanning electron microscopy (SEM) at different magnifications (500×, 1500×, 3000×, and 13,000×), as shown in Figure 2. The low-magnification images (Figure 2A) revealed a smooth, compact, and uniform surface with the transparency of plastic, free from noticeable holes, folds, or cracks, indicating good compatibility among the materials used in the preparation of LBDMs. The higher-magnification images (Figure 2B–D) displayed small particles on the film surface, likely resulting from the agglomeration of polymer materials forming small, aggregated structures. Additionally, during the drying process, small molecular substances may have volatilized first, with macromolecular substances continuously precipitating and depositing on the film surface.

#### 3.2.2. Wettability Analysis of LBDMs 

**Figure 3:**
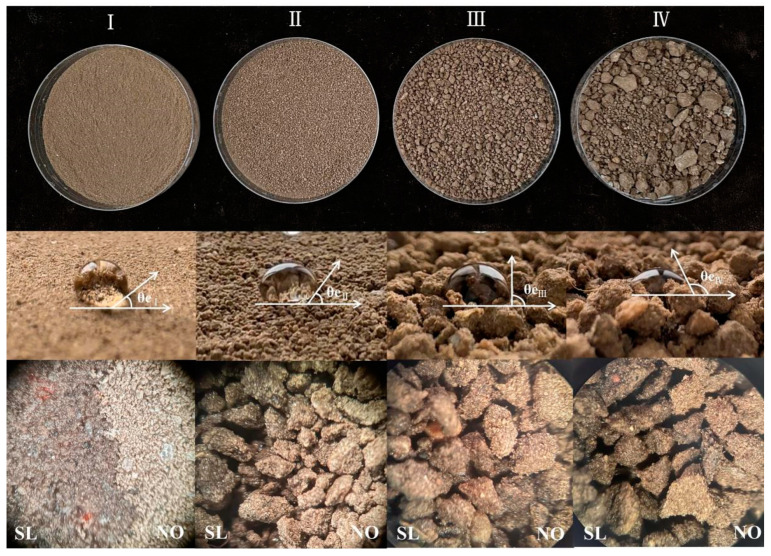
The wettability of LBDMs on soil surfaces with different particle sizes. (I) Grinding soil after passing through a 100-mesh sieve. (II) Soil passing through a 100-mesh sieve. (III) Conventional soil with large particles removed. (IV) Untreated conventional soil. (SL) Microscopic images of the soil surface sprayed with LBDMs (mag = 40×). (NO) Microscopic images of the soil surface without LBDMs application (mag = 40×).

To analyze the wettability of LBDMs, contact angle tests were conducted. The contact angles of LBDM droplets on soil surfaces with different particle sizes are shown in Figure 3. As the soil particle size increased, the contact angle also increased (θe_I_ = 37.5°, θe_II_ = 52.0°, θe_III_ = 90.0°, θe_IV_ = 116.2°), and the complete infiltration time of the film lengthened (T_I_ = 24 s, T_II_ = 6 s, T_III_ = 4 s, T_IV_ = 3 s). These results indicated a good wettability effect on soil surfaces of varying particle sizes. It was also observed in the experiment that, as the soil particle size became smaller, the soil surface layer after spraying LBDMs was more likely to form a film layer. This phenomenon confirmed that, under uniform soil flatness, reducing soil granularity and enhancing the binding force between soil aggregates made it easier to form a continuous film on the soil surface, significantly improving the film-forming effect.

**Figure 4:**
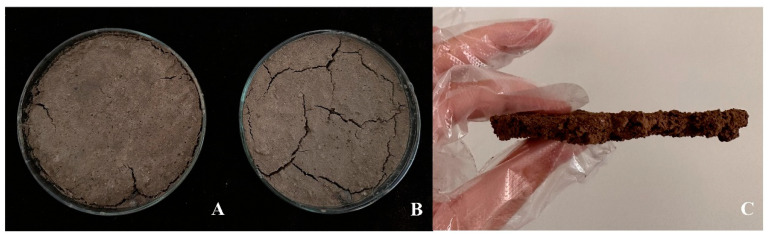
The film-forming effect of LBDMs on the soil surface. (**A**) Comparison of the soil surface with LBDM mulching after 7 days of water soaking. (**B**) Comparison of the soil surface without LBDM mulching after 7 days of water soaking. (**C**) Digital images of the soil cross-section after spraying LBDM mulching film.

LBDMs demonstrated excellent wettability, effectively covering soil surfaces with various particle sizes, bonding soil particles, and forming film-covered soil surface layers (Figure 4C). This good film-forming property is crucial for its warming and moisturizing effects. When examining the impact of simulated rainwater immersion on LBDMs’ stability (Figure 4B), many dry cracks of varying depths were observed on the surface of soil without LBDMs mulching. In contrast, the soil surface with LBDM mulching had a relatively uniform structure with only a few small cracks (Figure 4A). Soil cracks result from rapid water evaporation. Compared to the untreated soil sample, fewer cracks appeared on the LBDM-covered soil surface after rain immersion due to the protective layer formed by LBDMs. This layer slows water evaporation, reduces the agglomeration and shrinkage of soil aggregates, and thus minimizes the occurrence of surface cracks. These results further indicate that LBDMs effectively enhance the bonding force between surface soil aggregates, maintain soil granular structure stability, prevent soil moisture evaporation, and improve soil water stability and moisture conservation.

#### 3.2.3. Degradation Performance of LBDMs

**Figure 5:**
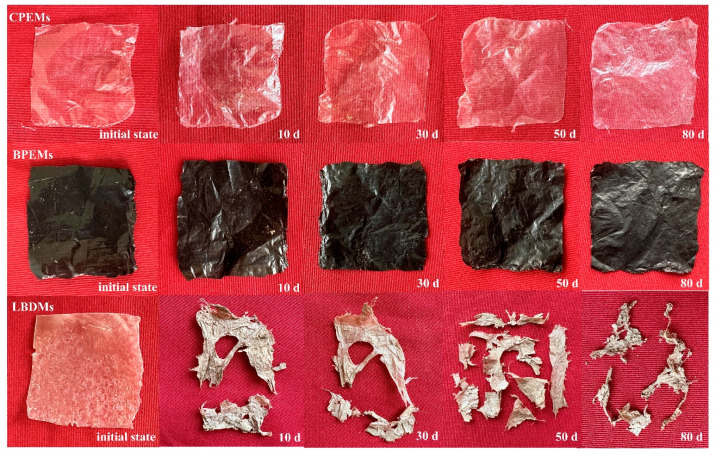
The surface morphology of CPEMs, BPEMs, and LBDMs after degradation at different soil burial times.

**Figure 6:**
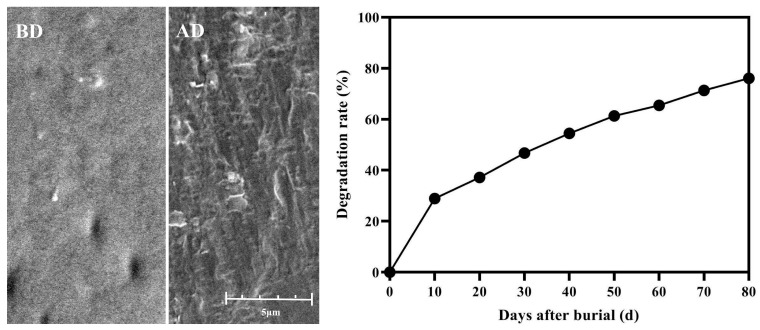
SEM images of the LBDMs’ surface morphology (mag = 13,000×) and the correlation between the degradation rate of LBDMs and soil burial duration. BD = before degradation (0 days after burial); AD = after degradation (80 days after burial).

The degradation of the film in the natural environment is influenced by seasonal and location-specific factors, such as humidity, temperature, sunlight, and microorganisms [39]. These factors compromise the film’s internal structure, leading to a loss of rigidity and toughness. Some researchers consider the weight loss method as a quantitative indicator of the degradation performance of films, with an increased degradation rate over time proving the degradability of LBDMs [40]. As shown in Figure 5, LBDMs softened and thinned after ten days of burial, with observable rupture and degradation. The LBDMs lost their original appearance and structural integrity during the degradation process, developed many holes and cracks, and gradually decomposed into small fragments. SEM images revealed that the surface morphology of the degraded film sample became wrinkled and uneven, with visible fragmented structures protruding from the surface (Figure 6). We also found that the degradation rate increased over time (Figure 6). After 50 days of burial in the soil, the film’s degradation rate exceeded 50%, classifying it as a biodegradable material [41]. Specifically, the degradation rate of LBDMs was 61.33% after 50 days and reached 76.09% after 80 days of burial, indicating that LBDMs have good degradability. These results demonstrated that LBDMs were one kind of biodegradable and environmentally friendly material.

### 3.3. Soil Temperature and Moisture

**Figure 7:**
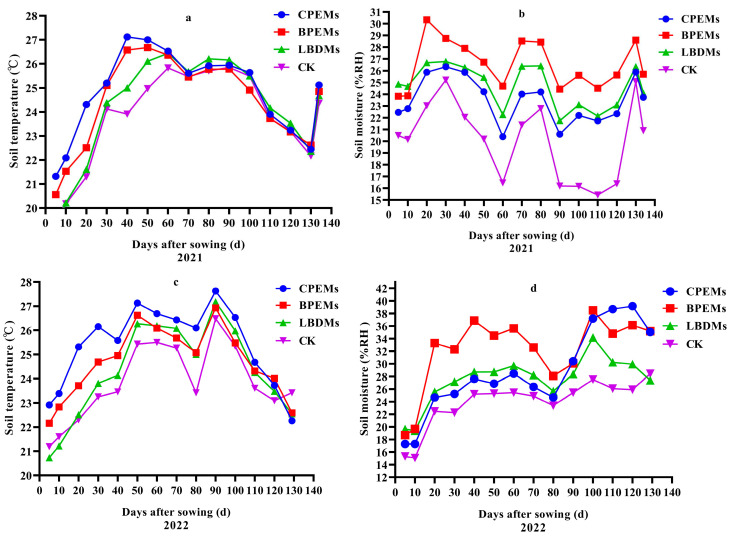
Soil temperature (**a**,**c**) and moisture (**b**,**d**) in the 0–10 cm layer under CPEMs, BPEMs, LBDMs, and CK treatments during the growing seasons of 2021 and 2022.

Film mulching significantly impacts the soil microenvironment, with soil temperature and moisture levels affecting crop yield [3]. Warmer soil and higher soil moisture content are positively related to peanut-seedling emergence and phenological development, canopy formation, radiation use efficiency, and pod yield. From sowing to harvest, the trend of soil temperature and moisture during the experimental period is shown in Figure 7. Compared to the control (CK), LBDMs demonstrated a more remarkable warming effect, raising the average temperature over two years by 0.56 °C. However, this warming effect was less pronounced than that of CPEMs and BPEMs, which increased temperatures by 1.21 °C and 0.72 °C, respectively. These findings align with the results of Sun et al. [42] and Sartore et al. [43]. In the later stages of crop growth, the difference in soil temperature among the treatments diminished compared to the earlier stages. This may be attributed to the crop canopy affecting the soil’s heat absorption from solar radiation, thus influencing soil temperature. Several studies have shown that the warming effect of mulching decreased in the later growth stages, as the plant canopy became fully established, narrowing the soil temperature gap between mulching and non-mulching treatments [44,45].

Soil moisture is a critical physical property of soil, playing a vital role in crop growth. Film mulching forms a barrier between the soil and the atmosphere, preventing soil moisture loss [10,46]. During the 2021 and 2022 growth stages, significant differences in soil moisture at a 10 cm depth were observed under different mulching conditions. LBDMs exhibited better soil moisture retention than CK, increasing average humidity by 19.25%. In comparison, CPEMs increased average humidity by 20.09%, while BPEMs showed the highest moisture retention, with a 35.75% increase over CK. This difference from soil temperature trends can be explained by the fact that increased soil temperature not only accelerates surface moisture loss but also promotes root development, enhances crop aboveground growth, and increases leaf transpiration. Excessive transpiration is not conducive to soil moisture retention [47]. Additionally, Chen et al. [48] found that higher soil moisture increased heat capacity and slowed temperature rise. This study could confirm the above point that LBDMs and BPEMs retained soil moisture better than CPEMs, though their soil temperature preservation was not as effective as that of CPEMs.

### 3.4. Effects of mulching films on Peanut Seedling Emergence

The seedling emergence rate is a crucial factor in determining yield. We recorded daily seedling emergence data. As shown in Table 1, all mulching treatments promoted peanut emergence and increased the emergence rate. Among them, BPEMs and CPEMs had the most significant effect, followed by LBDMs. In both 2021 and 2022, compared to CK, the LBDMs treatment advanced the peanut seedling stage by 4-to-6 days and effectively increased the emergence rate by 16.88% and 14.93%, respectively. It was found that increased soil temperature could improve seed germination and emergence [49]. At the seedling stage, the plant canopy was small, which allowed most of the film-mulched area to receive solar energy and the soil temperature to warm up. In addition, the water underneath the film could reduce the longwave radiation, which reduces the rate of decrease in soil temperature at night. Therefore, the diurnal temperature fluctuation in this stage involved faster warming up of mulched than un-mulched soil during the day and slower cooling at night, producing a mini-greenhouse effect.

### 3.5. Effects of Mulching Films on Peanut Growth Parameters

**Figure 8:**
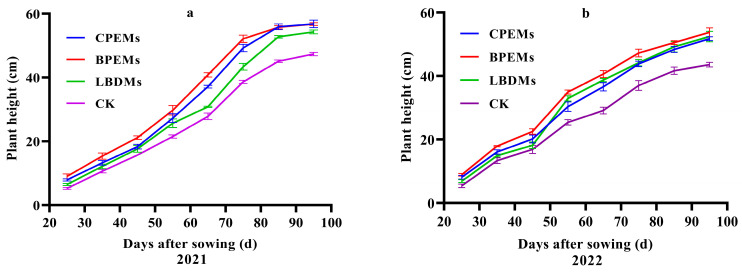
The trend in peanut plant height under CPEM, BPEM, LBDM, and CK treatments in 2021 (**a**) and 2022 (**b**).

**Figure 9:**
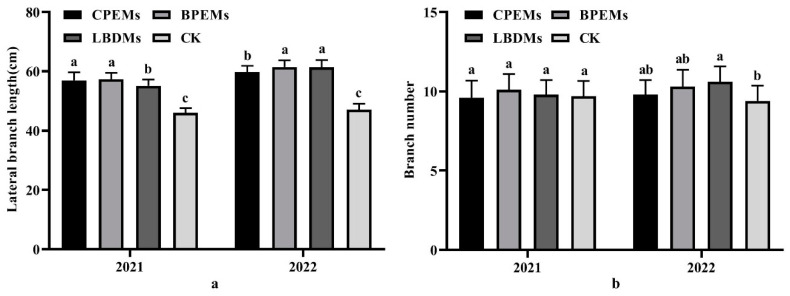
Lateral branch length (**a**) and branch number (**b**) of peanut plants at harvest period under CPEM, BPEM, LBDM, and CK treatments in 2021 and 2022. Values followed by different lowercase letters in the same column are significantly different among treatments at 0.05 level for the same factor.

To analyze the agronomic traits of peanuts, we recorded the plant height, lateral branch length, and number of branches. Based on two years of field data, the plant height of peanuts under different treatments showed a gradual increase over time (Figure 8). Compared to CK, mulching with LBDMs significantly increased plant height throughout the growth period. Additionally, the lateral branch length and number of branches mulched with LBDMs resulted in longer lateral branch lengths and more branches than CK and comparable to or even better than CPEMs and BPEMs treatments (Figure 9).

In 2021, the average length of lateral branches mulched with LBDMs was 55.15 cm, which was 9.2 cm longer than CK, but 1.8 cm and 2.2 cm shorter than CPEMs and BPEMs, respectively (Figure 9a). The mean number of branches for LBDM treatment was 9.8, which is not significantly different from CK, CPEMs, and BPEMs (*p* < 0.05, Figure 9b). Similar results were observed in 2022, where the average length of lateral branches mulched with LBDMs was 61.35 cm, 14.3 cm longer than CK, and 1.55 cm longer than CPEMs, with no significant differences from BPEMs (*p* < 0.05, Figure 9a). The mean number of branches for LBDMs treatment in 2022 was 10.6, which was 1.2 more than that of CK and not significantly different from that of CPEMs and BPEMs (*p* < 0.05, Figure 9b). Favorable soil temperature plays a crucial role in optimal plant growth. These findings confirm that LBDM mulching promotes the early growth of peanut plants, resulting in faster seedling emergence and better overall plant growth. This phenomenon can be attributed to film mulching, which alters soil temperature and moisture to conditions more suitable for seedling emergence and plant growth. This conclusion aligns with the reports of Wang et al. [50] and Sun et al. [42].

### 3.6. Effects of Mulching Films on Peanut Pod Traits

To assess the effects of LBDM mulching treatments on peanut pod quality, we recorded the full pod rate, immature pod rate, kernel rate, single kernel rate, double kernel rate, and germinated kernel rate. The results in Table 2 show that the full pod rate, kernel rate, and germinated kernel rate for LBDM mulching treatments were significantly higher than those for CK (*p* < 0.05), while the immature pod rate was lower. Compared to the CPEM and BPEM treatments, the germination rate of LBDMs was lower, and the full pod rate and kernel rate were equivalent to that of CPEMs, equivalent to or slightly worse than that of BPEMs. In addition, there were no significant differences among the four treatments regarding the single kernel rate and double kernel rate.

In 2021, the full pod rate of peanuts under the LBDM treatment was 72.73%, which was 6.04% higher than that of CK, 3.93% lower than that of BPEMs, and not significantly different from that of CPEMs (*p* < 0.05). The immature pod rate for LBDMs was 24.97%, 5.60% lower than that of CK, 3.91% higher than that of BPEMs, and not significantly different from that of CPEMs (*p* < 0.05). The kernel rate for LBDMs was 73.40%, 1.97% lower than that of BPEMs, and not significantly different from that of CK and CPEMs (*p* < 0.05). The single and double kernel rates for LBDMs were 21.37% and 77.06%, respectively, with no significant differences from the other treatments (*p* < 0.05). The germinated kernel rate for LBDMs was 4.02%, 2.84% higher than that of CK, and 2.97% and 6.86% lower than that of CPEMs and BPEMs, respectively. The results for 2022 showed some differences from 2021. The full pod rate under LBDM treatment was 67.67%, 7.23% higher than that of CK, 5.74% lower than that of BPEMs, and not significantly different from that of CPEMs (*p* < 0.05). The immature pod rate for LBDMs was 29.37%, with no significant differences from the other treatments (*p* < 0.05). The kernel rate for LBDMs was 83.09%, 12.88% higher than that of CK, and not significantly different from that of CPEMs and BPEMs (*p* < 0.05). The single and double kernel rates for LBDMs were 22.61% and 75.93%, respectively, with no significant differences from the other treatments (*p* < 0.05). The germinated kernel rate for LBDMs was 0.74%, 7.64%, and 2.85% lower than that if BPEMs and CPEMs, respectively, and not significantly different from CK (*p* < 0.05).

### 3.7. Effects of Mulching Films on Peanut Yield

To explore the effect of LBDMs treatment on yield, we studied productivity per plant, 100-pod weight, and 100-kernel weight. Over two years, we recorded peanut yield and confirmed that mulching with LBDMs could significantly affect yield. The data in Table 3 indicate that mulching with LBDMs, CPEMs, and BPEMs significantly increased pod yield compared to CK.

In 2021, the yield increase rate for the LBDM mulching treatment was 8.42%, which was 2.49% and 6.2% lower than that of CPEMs and BPEMs, respectively. In 2022, the yield increase rate for LBDMs was 20.25%, 5.98% higher than that of CPEMs and 3.11% lower than that of BPEMs. Overall, the average yield increase rate over the two years of LBDMs mulching treatment was significantly better than that of CPEMs but not as good as that of BPEMs. In 2021, the productivity per plant and 100-pod weight for LBDMs were 19.38 g and 245.00 g, respectively, which were 2.30 g and 11.00 g higher than CK’s values but not significantly different from the values of CPEMs and BPEMs (*p* < 0.05). There was no significant difference in 100-kernel weight among the four treatments (*p* < 0.05). In 2022, the productivity per plant for LBDMs was 20.67 g, which was 3.74 g and 2.45 g higher than that of CK and CPEMs, respectively, but not significantly different from that of BPEMs (*p* < 0.05). The 100-pod weight for LBDMs was 242.08 g, which was 6.55 g higher than that of CK, with no significant differences from that of CPEMs and BPEMs (*p* < 0.05). The 100-kernel weight for LBDMs was 95.47 g, which was 6.55 g and 6.37 g higher than that of CK and CPEMs, respectively, but not significantly different from that of BPEMs (*p* < 0.05).

The pod yield in the mulched treatments, including the LBDM-mulched treatments, was higher than that in CK. Similar results were reported by Waterer [51]. Film mulching increases soil temperature by several degrees, promoting better growth during the early growth period and more water absorption in the later period. Notably, LBDM mulching improved plant height, lateral branch length, number of branches, and dry matter accumulation in individual plants and increased productivity per plant, full pod rate, kernel rate, 100-pod weight, and 100-kernel weight, leading to an increased pod yield. This result aligns with Song’s report [22]. Compared with CK, the pod yield, productivity per plant, 100-pod weight, 100-kernel weight, full pod rate, and kernel rate of LBDMs were better, but it did not affect pod traits such as the single kernel rate. This may be due to film mulching promoting peanut flower bud differentiation, increasing the number of effective flowers and needles, thereby promoting an increase in the number of pods per plant and increasing peanut yield [52]. Additionally, film mulching prevents pegs developing during later growth stages from entering the soil, thus conserving nutrients for developing pods set earlier, increasing the number of full pods, and reducing the number of immature pods [53,54].

The productivity per plant, 100-pod weight, and 100-kernel weight of LBDMs treatment was significantly better than that of CK and equivalent to or even better than PE mulching treatments. However, in terms of pod yield, the LBDM treatment’s result was significantly higher than that of the CK but lower than that of the BPEM, possibly due to the emergence rate. This finding is consistent with the research of Kunzova et al. [55], which highlighted that seedling emergence and establishment are key processes in grain yield determination.

## 4. Conclusions

Overall, this study underscores the potential of LBDMs as environmentally friendly alternatives to traditional PE films. LBDMs demonstrated good degradability and wettability and can be simply sprayed onto the soil to form a film layer to serve as agriculture mulching, which is highly facile and efficient as compared with traditional PE films. It optimizes the soil environment, promoting peanut seed germination and root development, supporting healthy plant growth, and laying the foundation for increased peanut yield. These improvements were comparable to those achieved with ordinary PE films and even outperformed in regard to some aspects of crop growth and yield. In general, LBDMs not only provide the heat- and moisture-retention benefits of PE films, advancing the seedling stage, increasing the emergence rate, promoting crop growth and development, accelerating the growth process, and increasing yield, but also reduce labor input and residual soil pollution due to their simple application and degradation performance. Therefore, promoting the use of LBDMs as a substitute for ordinary PE films in agricultural production is of great significance. However, further studies on economic cost, field application characteristics, and supporting spraying equipment are necessary.

## Figures and Tables

**Table 1:** Seedling emergence analysis in 2021 and 2022.

Year	2021			2022		
Treatments	Seedling Stage		Emergence Rate (%)	Seedling Stage		Emergence Rate (%)
	Date	DAS		Date	DAS	
CPEMs	15 May	12	87.66 ± 3.52 b	19 May	11	89.52 ± 3.29 a
BPEMs	15 May	12	91.35 ± 2.66 a	18 May	10	92.17 ± 4.29 a
LBDMs	19 May	16	82.59 ± 2.28 c	23 May	15	81.08 ± 3.10 b
CK	25 May	22	65.71 ± 2.36 d	27 May	19	66.15 ± 3.69 c

Note: Data are presented as mean ± SE, *n* = 3. Values followed by different lowercase letters in the same column are significantly different among treatments at 0.05 level for the same factor. Seedling stage: the time required for 50% of seedlings to emerge and expand their first true leaf; DAS, days after sowing.

**Table 2:** Effect of mulching on peanut pod quality in 2021 and 2022.

	Full Pod Rate (%)	Immature Pod Rate (%)	Kernel Rate (%)	Single Kernel Rate (%)	Double Kernel Rate (%)	Germinated Kernel Rate (%)
Year	2021					
CPEMs	72.31 ± 0.96 b	25.24 ± 1.18 b	74.02 ± 1.42 ab	21.27 ± 1.40 a	77.59 ± 1.85 a	6.96 ± 0.92 b
BPEMs	76.66 ± 1.75 a	21.06 ± 0.92 c	75.37 ± 0.86 a	20.87 ± 0.83 a	78.29 ± 0.97 a	10.88 ± 1.30 a
LBDMs	72.73 ± 1.13 b	24.97 ± 0.64 b	73.40 ± 1.00 b	21.37 ± 0.89 a	77.06 ± 1.38 a	4.02 ± 0.58 c
CK	66.69 ± 1.37 c	30.57 ± 1.59 a	73.32 ± 1.06 b	21.68 ± 1.22 a	77.74 ± 1.25 a	1.18 ± 0.62 d
Year	2022					
CPEMs	66.02 ± 2.23 bc	31.13 ± 2.65 ab	81.32 ± 1.54 a	24.91 ± 1.72 a	72.99 ± 1.82 a	3.59 ± 0.28 ab
BPEMs	73.41 ± 3.36 a	25.11 ± 2.68 b	80.59 ± 4.67 a	20.98 ± 4.18 a	74.48 ± 4.74 a	8.38 ± 2.63 a
LBDMs	67.67 ± 2.58 b	29.37 ± 3.91 ab	83.09 ± 7.86 a	22.61 ± 1.01 a	75.93 ± 0.29 a	0.74 ± 0.82 c
CK	60.44 ± 3.20 c	36.52 ± 4.23 a	70.21 ± 2.76 b	26.34 ± 3.68 a	71.59 ± 4.85 a	1.25 ± 1.44 bc

Data are presented as mean ± SE, *n* = 3. Values followed by different lowercase letters in the same column indicate significant differences among treatments at the 0.05 level for the same factor.

**Table 3:** Effect of mulching on peanut yield and yield components in 2021 and 2022.

	Pod Yield (kg·ha^−1^)	Yield Increase Compared to CK (%)	Productivity per Plant (g)	100-Pod Weight (g)	100-Kernel Weight (g)
Year	2021				
CPEMs	4975.00 ± 131.05 ab	10.19	18.43 ± 0.49 ab	238.63 ± 5.47 ab	97.24 ± 2.55 a
BPEMs	5175.00 ± 119.06 a	14.62	20.37 ± 0.97 a	247.33 ± 2.25 a	97.15 ± 1.82 a
LBDMs	4895.00 ± 129.33 bc	8.42	19.38 ± 1.04 a	245.00 ± 1.80 a	96.80 ± 1.04 a
CK	4515.00 ± 83.52 c	-	17.08 ± 0.96 b	234.00 ± 5.00 b	96.00 ± 0.98 a
Year	2022				
CPEMs	4965.00 ± 70.53 b	14.27	18.22 ± 1.07 b	239.99 ± 2.54 bc	89.10 ± 2.32 b
BPEMs	5360.00 ± 106.42 a	23.36	21.24 ± 0.41 a	246.45 ± 3.65 a	96.23 ± 3.29 a
LBDMs	5225.00 ± 92.60 a	20.25	20.67 ± 0.61 a	242.08 ± 0.68 ab	95.47 ± 1.14 a
CK	4345.00 ± 102.10 c	-	16.93 ± 0.60 b	235.53 ± 1.60 c	88.92 ± 4.20 b

Data are presented as mean ± SE, *n* = 3. Values followed by different lowercase letters in the same column indicate significant differences among treatments at the 0.05 level for the same factor.

## Data Availability

Data are contained within the article.
